# The Emerging Picture of the Roles of CircRNA-CDR1as in Cancer

**DOI:** 10.3389/fcell.2020.590478

**Published:** 2020-12-01

**Authors:** Chaohua Jiang, Xiaohong Zeng, Renfeng Shan, Wu Wen, Jianfeng Li, Jinfeng Tan, Lei Li, Renhua Wan

**Affiliations:** ^1^Department of General Surgery, The First Affiliated Hospital of Nanchang University, Nanchang University, Nanchang, China; ^2^Imaging Department, The First Affiliated Hospital of Nanchang University, Nanchang University, Nanchang, China

**Keywords:** circular RNA, CDR1as, cancer, biomarker, miRNA sponge

## Abstract

Circular RNAs (circRNAs) are covalently closed circular structures without 5′ caps and 3′ tails, which are mainly formed from precursor mRNAs (pre-mRNAs) via back-splicing of exons. With the development of RNA sequencing and bioinformatic analysis, circRNAs were recently rediscovered and found to be widely expressed in the tree of life. Cerebellar degeneration-related protein 1 antisense RNA (CDR1as) is recognized as one of the most well-identified circRNAs. It contains over 70 miR-7 binding sites and can regulate gene activity by sponging miR-7. Increasing numbers of studies have recently demonstrated that CDR1as is abnormally expressed in many types of tumors, such as colorectal cancer, cholangiocarcinoma and osteosarcoma, and plays a vital role in the development of cancer. However, there are few reviews focusing on CDR1as and cancer. Hence, it is important to review and discuss the role of CDR1as in cancer. Here, we first review the main biological features of CDR1as. We then focus on the expression and roles of CDR1as in cancer. Finally, we summarize what is known on the role of CDR1as in cancer and discuss future prospects in this area of research.

## Introduction

Circular RNAs (circRNAs) are covalently closed circular structures without 5′ caps and 3′ tails, which are mainly formed from precursor mRNAs (pre-mRNAs) via the back-splicing of exons (Hansen et al., 2013a; Jeck et al., 2013; Memczak et al., 2013; Jeck and Sharpless, 2014; Lasda and Parker, 2014). The electron-microscopic discovery of circular RNAs in an RNA virus was first reported in Sanger et al. (1976). With the development of RNA sequencing and bioinformatic analysis, circRNAs were recently rediscovered and found to be widely expressed in the tree of life (Wang et al., 2010, 2014; Salzman et al., 2013). Furthermore, the expression of circular RNAs is cell type- and tissue-specific (Barrett and Salzman, 2016; Qian et al., 2020; Zhang S. et al., 2020), implying that the expression of circular RNAs is influenced by the specific cellular environment. As circRNAs lack 3′ or 5′ tails, they are more resistant to degradation by exonuclease RNase R and have longer half-lives than associated linear mRNAs (Chen, 2016; Patop and Kadener, 2018; Shang et al., 2019), which indicated that circRNAs could be more easily detectable biomarkers.

CircRNAs exert their function through several mechanisms (Geng et al., 2018; Patop and Kadener, 2018; Zhou et al., 2018; Arnaiz et al., 2019). Firstly, circRNAs can function as miRNA sponges and competitive endogenous RNAs to modulate the activity of miRNAs by competing for miRNA-binding sites (Salmena et al., 2011; Hansen et al., 2013a; Rong et al., 2017; Dori and Bicciato, 2019; Verduci et al., 2019; Lin et al., 2020; Liang et al., 2020). Secondly, some circRNAs can bind and interact with transcription factors to regulate the transcription of targeted gene (Memczak et al., 2013; Braunschweig et al., 2014). For example, ci-Ankyrin Repeat Domain 52 (ci-ANKRD52) can bind to the Pol II elongation complex and in result the accumulation of elongation Pol II at transcriptional sites to promote the transcription of ANKRD52 (Zhang et al., 2013). Thirdly, circRNAs can bind to target proteins to influence their decay or accumulation (Schneider et al., 2016; Abdelmohsen et al., 2017). For example, circ-Foxo3 can promote MDM2-induced p53 ubiquitination by binding to both NDM2 and p53 protein (Du et al., 2017).

Cerebellar degeneration-related protein 1 antisense RNA (CDR1as), also known as circular RNA sponge for miR-7 (ciRS-7) (Memczak et al., 2013), is recognized as one of the most well-identified circRNAs (Hansen et al., 2011, 2013a). As there are over 70 miR-7 binding sites in CDR1as, it acts as a miR-7 sponge and regulates the latter’s activity (Hansen et al., 2011, 2013a; Yao et al., 2018). Additionally, CDR1as is globally co-expressed with miR-7 in the brain, indicating that ciRS-7 may be a better term for it. However, none of the 70 identified binding sites of miR-7 in CDR1as are complementary with the entire miR-7 sequence, and only match the 5 end “seed region” of miR-7 (Guo et al., 2020). Recently, there is increasing evidence that CDR1as is overexpressed in many tumor types, such as colorectal cancer (Tang et al., 2017), cholangiocarcinoma (Jiang et al., 2018) and osteosarcoma (Xu et al., 2018), and plays a vital role in the development of cancer. However, there are few reviews focusing on CDR1as and cancer. Hence, it is important to review and discuss the role of CDR1as in cancer.

## The Biogenesis of CDR1as

**FIGURE 1:**
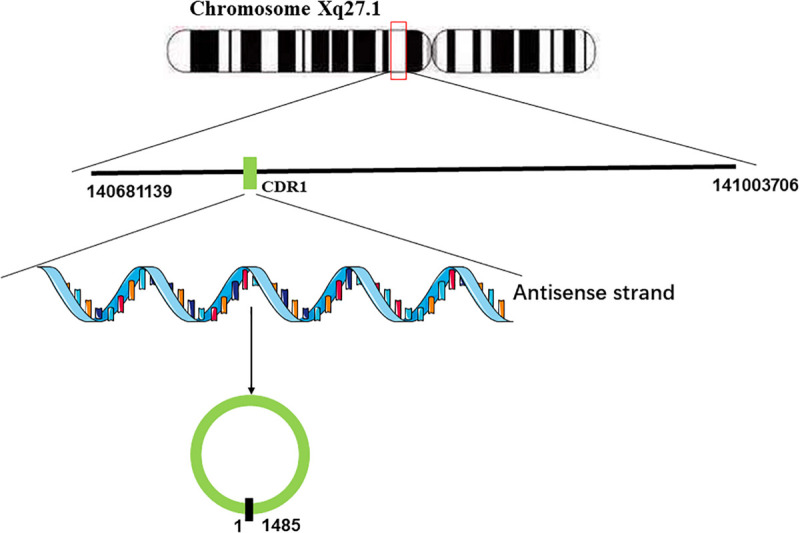
Generation of CDR1as. CDR1 is located on Chromosome Xq27.1. The antisense strand of CDR1 undergoes back-splicing of the 5′ and 3′ ends to form the circular RNA.

Hansen et al. (2011) found that miR-671 could decrease the levels of CDR1 mRNA by inducing the cleavage of the natural antisense transcript (NAT) of CDR1 mediated by argonaute (AGO)-2. They used 3 Rapid amplification of cDNA ends (RACE) analyses to characterize it and failed to find any polyadenylation, which implied that there is no poly(A)-tail in CDR1 NAT (Hansen et al., 2011). Furthermore, CDR1 NAT was found to be resistant to nicotinamide pyrophosphatase and terminal 5-phosphate-dependent exonuclease, implying that there is also no 5′-terminal cap in the natural antisense transcript (NAT) of CDR1 (Hansen et al., 2011). Taken together, these results indicated that the natural antisense transcript (NAT) of CDR1 is a circular RNA. Generally, the formation of circular RNAs is often facilitated by flanking inverted repeats of the primate-specific Alu elements (Chen, 2016; Li et al., 2018a). However, the gene locus of CDR1as lacks Alu elements, indicating that there must be another mechanism of CDR1 NAT circularization. Barrett et al. found the promoter of LINC00632 could promote the expression of CDR1as, and the LINC00632 locus contains the CDR1as sequence (Barrett et al., 2017). According to their research, it stands to reason that the pre-linear RNA of CDR1as transcribed by the antisense strand of CDR1 concomitantly undergoes back-splicing of the 5′ and 3′ ends to form the circular RNA (Barrett et al., 2017; Figure 1).

## The Biological Functions of CDR1as

CDR1as is a closed circular RNA formed from the antisense transcript of the cerebellum degeneration-related antigen 1 (CDR1) gene, whose length is 1,485 bp (Hansen et al., 2013b). CDR1as is highly expressed in the human brain and plays a vital role in midbrain development (Memczak et al., 2013), which might account for its relationship with neurodegenerative diseases, such as Alzheimer and Parkinson’s disease (Shi et al., 2017; Azari et al., 2020). And the overexpression of CDR1as in zebrafish resulted in defects in the midbrain region similar to the knockdown of miR-7 (Memczak et al., 2013). At the same time, CDR1as was found to regulate the transcription and secretion of insulin by sponging miR-7 in pancreatic islet cells (Xu et al., 2015), which might be a potential therapeutic target for diabetes. As CDR1as can regulate the activity of miR-7 by sponging it, CDR1as exerts a wide range of physiological and pathological effects (Li et al., 2018b). Accordingly, CDR1as plays an important role in cellular proliferation and differentiation as well as cancer invasion and metastasis (Yan et al., 2018; Li L. et al., 2019; Wang et al., 2019; Yang L. et al., 2019; Zhou et al., 2019; Zou et al., 2019; Chen et al., 2020; Hanniford et al., 2020; Yang et al., 2020; Zhao F. et al., 2020).

## CDR1as Functions as a miRNA Sponge

There is increasing evidence that CDR1as can act as an miRNA sponge by absorbing several miRNAs (Shi et al., 2017; Kyei et al., 2020). Among these miRNAs, miR-7 functions as a tumor suppressor in many cancer types, such as osteosarcoma, breast cancer, hepatocellular carcinoma, and colorectal cancer (Yu et al., 2016; Tang et al., 2017; Yang X. et al., 2017; Xu et al., 2018). CDR1as was found to promote the proliferation and metastasis of cancer cells by sponging miR-7 (Tang et al., 2017; Xu et al., 2018; Yang L. et al., 2019). Furthermore, since conserved miR-7 target sites on CDR1as are complementary to miR-7, these sites could be a lodging site for transport (Memczak et al., 2013; Piwecka et al., 2017). Furthermore, CDR1as was able to sponge miR-1270 and modulate the activity of miR-1270, resulting in drug resistance, proliferation and metastasis of cancer (Su et al., 2019; Yuan et al., 2019; Zhao et al., 2019). Similarly, CDR1as was found to promote the progression of cholangiocarcinoma and osteoarthritis by sponging miR-641 (Li D. et al., 2020; Zhang W. et al., 2020). Moreover, CDR1as was reported to sponge miR-135b-5p, miR-219a, miR-1299, and miR-876-5p in ovarian cancer, non-small-cell lung cancer and esophageal squamous cell carcinoma, respectively (Sang et al., 2018b; Chen et al., 2019; Cai et al., 2020; Li Y. et al., 2020; Meng et al., 2020). In addition, CDR1as was found to stimulate tube formation in microvascular endothelial cells by decreasing the expression of miR-26a-5p (Cui et al., 2020). Taken together, these studies demonstrate that CDR1as plays varied roles in the occurrence and development of cancer and might be a potential therapeutic target.

## The Expression of CDR1as in Cancer

Many studies have demonstrated that CDR1as is expressed abnormally in many cancer types (Pan et al., 2018; Su et al., 2018; Tanaka et al., 2019; Lin et al., 2020; Tian et al., 2020; Zhou et al., 2020). The expression of CDR1as is upregulated in most tumors, such as colorectal cancer, hepatocellular carcinoma and breast cancer, and it exerts a tumor-promoting effect (Tang et al., 2017; Weng et al., 2017; Xu et al., 2017; Yang X. et al., 2017; Yuan et al., 2019; Zhang Z. et al., 2020; Zhao Y. H. et al., 2020). Especially in colon cancer, Kristensen et al. (2020) found that CDR1as is absent in the cancer cells, but highly expressed in stromal cells within the tumor microenvironment. Similarly, CDR1as showed low expression in other tumors, such as ovarian cancer, melanoma and bladder cancer, indicating that it functions as a tumor suppressor (Chen et al., 2019; Zhao et al., 2019). These findings indicate that CDR1as can function either as a tumor suppressor or promoter in different tumor microenvironments, which will be discussed below (Table 1).

**TABLE 1:** The expression and roles of circ-CDR1as in different human cancers.

**Cancer type**	**Expression**	**Functional roles**	**Related signaling pathways**	**References**
Melanoma	Downregulated	Migration	CDR1as-IGF2BP3	Sun et al., 2016; Zhang L. et al., 2018; Hanniford et al., 2020
Non-small-cell lung cancer	Upregulated	Proliferation Migration Apoptosis	CDR1as-219a-5p/SOX5	Zhang X. et al., 2018; Li Y. et al., 2020
Esophageal squamous cell carcinoma	Upregulated	Proliferation Migration Autophagy	CDR1as-miR-1299-EGFR CDR1as-miR-7-KLF4 CDR1as-miR-7-HOXB13 CDR1as-miR-876-5p-MAGE-A	Li R. C. et al., 2018; Sang et al., 2018b; Huang et al., 2019; Meng et al., 2020
Colorectal cancer	Upregulated	Proliferation Migration	CDR1as-miR-7/HOXB13	Tang et al., 2017; Weng et al., 2017; Li R. C. et al., 2018
Hepatocellular carcinoma	Upregulated	Proliferation Migration	CDR1as-miR-7	Yu et al., 2016; Xu et al., 2017; Yang X. et al., 2017; Su et al., 2019
Nasopharyngeal carcinoma	Upregulated	Proliferation	CDR1as—miR-7-E2F3	Zhong et al., 2019
Laryngeal squamous cell carcinoma	Upregulated	Proliferation Migration	CDR1as—miR-7-CCNE1 CDR1as-miR-7-PIK3CD	Zhang J. et al., 2018
Osteosarcoma	Upregulated	Proliferation	CDR1as-miR-7	Xu et al., 2018
Cholangiocarcinoma	Upregulated	Proliferation Migration	CDR1as-miR-641	Jiang et al., 2018; Li D. et al., 2020
Gastric cancer	Upregulated	Proliferation Migration Chemoresistance	CDR1as-miR-7-PTEN CDR1as-miR-135-TRPC1 CDR1as-miR-7-5p-REGgama	Pan et al., 2018; Li C. et al., 2019; Zhang Z. et al., 2020
Breast cancer	Upregulated	Proliferation Migration Chemoresistance	CDR1as—miR-1299-EGFR CDR1as-miR-7-REGγ CDR1as-miR-1299-MMPs	Sang et al., 2018a; Uhr et al., 2018; Yang et al., 2019a,b; Meng et al., 2020
Ovarian cancer	Downregulated	Proliferation-inhibition Migration-Inhibition Chemosensitivity	CDR1as-miR-135B-5P CDR1as-miR-1270-SCAI	Chen et al., 2019; Zhao et al., 2019
Bladder cancer	Downregulated	Proliferation-inhibition Migration-Inhibition Chemosensitivity	CDR1as-miR1270-APAF1 CDR1as-miR-135a	Li P. et al., 2018; Yuan et al., 2019

## The Roles of CDR1as in Cancer

### The Roles of CDR1as in Tumor Growth

**FIGURE 2:**
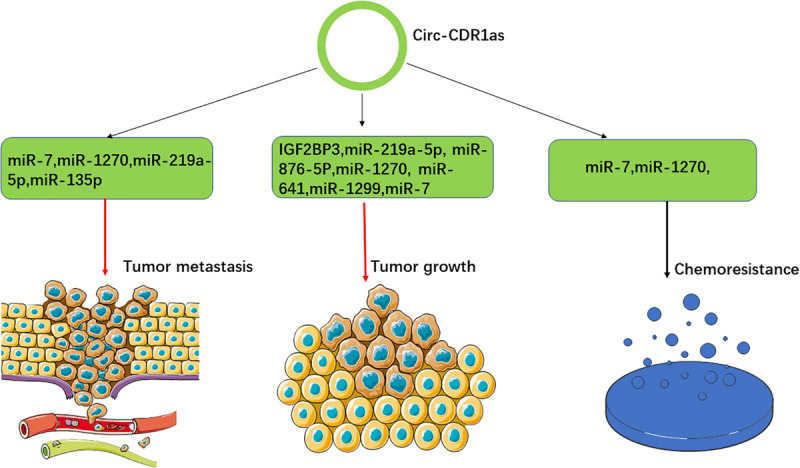
Overview of the roles of CDR1as in cancer. CDR1as exerts its roles in tumor growth, cancer metastasis and cancer chemoresistance via multiple signaling pathways.

Numerous studies have demonstrated that CDR1as is involved in the regulation of tumor growth by sponging several miRNAs and regulating multiple signaling pathways. For example, miR-7 acts as a tumor suppressor that can regulate cellular proliferation and various biological process by triggering the signal transduction of the growth factors (Sun et al., 2016; Yang Z. et al., 2017). CDR1as is upregulated in numerous cancers, where it can sponge miR-7 and stimulate the expression of the downstream targeted genes, such as E2F3, EGFR, IGF-1R, CCNE1, PIK3CD, RAF1, PTEN, and KLF4. Accordingly, CDR1as was found to promote tumor proliferation in nasopharyngeal carcinoma, colorectal cancer, non-small-cell lung cancer, osteosarcoma, laryngeal squamous cell carcinoma, gastric cancer and esophageal squamous cell carcinoma, respectively (Tang et al., 2017; Yang X. et al., 2017; Li R. C. et al., 2018; Pan et al., 2018; Xu et al., 2018; Zhang J. et al., 2018; Zhang X. et al., 2018; Huang et al., 2019; Zhong et al., 2019; Yang et al., 2020). These studies demonstrated that the CDR1as/miR-7 axis plays a vital role in tumor growth and might be a potential target for cancer therapy. AFP (alpha-fetoprotein), a biomarker of hepatocellular carcinoma, is the target gene of miR-1270. CDR1as can sponge miR-1270 and upregulate the expression of AFP to promote tumor growth, invasion and metastasis in hepatocellular carcinoma (Su et al., 2019). Similarly, the overexpression of CDR1as can stimulate tumor proliferation, invasion and metastasis by sponging miR-641 and stimulating the expression of AKT3 and mTOR in cholangiocarcinoma (Li D. et al., 2020). Furthermore, CDR1as knockdown inhibited tumor growth, invasion and metastasis by regulating the miR-219a-5p/SOX5 axis in non-small-cell lung cancer (Li Y. et al., 2020). Similarly, the knockdown of CDR1as could inhibit tumor growth via miR-135p in ovarian cancer (Chen et al., 2019). However, CDR1as was reported to inhibit tumor growth in glioblastoma multiforme by disrupting p53/MDM2 complex formation (Lou et al., 2020), which implies that CDR1as has different effects on cell growth in different cancer types (Figure 2).

### The Roles of CDR1as in Cancer Metastasis

Cancer metastasis is the critical step of cancer development and progression and many studies reported that CDR1as could influence cancer metastasis. As described above, CDR1as can promote cancer metastasis via multiple signaling pathways (Su et al., 2019; Li D. et al., 2020; Li Y. et al., 2020). In addition, CDR1as was found to interact with IGF2BP3 to promote tumor invasion and metastasis in melanoma (Zhang L. et al., 2018; Hanniford et al., 2020). Similarly, CDR1as could enhance the expression of MAGE-A family by sponging miR-876-5p to promote the progression of esophageal squamous cell carcinoma (Sang et al., 2018b). Furthermore, CDR1as could regulate miR-7/HOXB13 and miR-1299/MMPs to promote the metastasis of esophageal squamous cell carcinoma and triple-negative breast cancer, respectively (Li R. C. et al., 2018; Sang et al., 2018a). However, CDR1as was found to sponge miR-135b-5p and upregulate the expression of HIF1AN to inhibit the growth, invasion and metastasis of ovarian cancer (Chen et al., 2019), which shows that the roles of CDR1as in tumor metastasis vary in different cacer types (Figure 2).

### The Roles of CDR1as in Cancer Chemoresistance

Chemoresistance is the main obstacle to cancer therapy and remains a great challenge for improving the clinical outcomes of cancer patients (Zheng, 2017; Yeldag and Rice, 2018). A number of studies have demonstrated that the dysregulated expression of CDR1as is related to cancer chemoresistance (Uhr et al., 2018; Yang et al., 2019a,b; Mao and Xu, 2020). Two studies showed that the knockdown of CDR1as could increase the chemosensitivity of 5-fluorouracyl- and cisplatin-resistant breast cancer cells by sponging miR-7 (Yang et al., 2019a,b). Similarly, downregulation of CDR1as could modulate the miR-7-5p/REGγ axis to promote low-dose diosbulbin-B-induced gastric cancer cell death (Li C. et al., 2019). Furthermore, another study reported that CDR1as could regulate stemness and promote cisplatin chemoresistance in NSCLC cells by targeting the miR-641/HOXA9 axis (Zhao Y. et al., 2020). Taken together, these results indicate that CDR1as might be a potential therapeutic target for overcoming cancer chemoresistance. However, other studies demonstrated that the overexpression of CDR1as could increase the sensibility to cisplatin by sponging miR-1270 in bladder and ovarian cancer (Yuan et al., 2019; Zhao et al., 2019). The inconsistent results demonstrate that the roles of CDR1as in chemoresistance vary in different cancer types (Figure 2).

## Conclusion and Perspectives

CircRNAs were previously considered to be the products of faulty RNA splicing (Kristensen et al., 2018). However, with the development of RNA sequencing and bioinformatic analysis, circRNAs were recently rediscovered and found to be widely expressed in the tree of life (Wang et al., 2010, 2014; Salzman et al., 2013). Increasing numbers of studies demonstrated that circRNAs are abnormally expressed in cancer and exert a vital role in cancer progression through a complicated gene regulatory network. Here, we firstly systematically reviewed and discussed the roles of CDR1as in cancer, listing studies that might deepen our understanding of how it modulates cancer progression. Additionally, the relationship between CDR1as expression and clinicopathological characteristics was summarized. In detail, high CDR1as expression was associated with worse clinicopathological characteristics, including the T status, N status, histological grade, TNM stage and distant metastasis in solid tumors, such as esophageal squamous cell carcinoma (ESCC), non–small cell lung cancer (NSCLC), colorectal cancer (CC), and hepatocellular carcinoma (Zou et al., 2020). Numerous experiments have demonstrated that CDR1as might be an oncogene and promote cellular proliferation and cancer metastasis. In addition, the expression of CDR1as was found to be associated with poor prognosis in cancer patients. As circRNAs lack 3′ tails or 5′ caps, they are more resistant to degradation by exonuclease RNase R and have more long half-lives than associated linear mRNAs (Chen, 2016). Meantime, some studies reported that stably existed in human body fluids, such as serum, plasma, and saliva. Furthermore, CDR1as was reported to have a specificity of 74% in the diagnosis of digestive system-derived tumors (Zou et al., 2020). The AUC of the ROC curve represents the comprehensive accuracy rate of detection, and according to the results of this study, detection of CDR1as had an AUC of 0.84 (95% CI, 0.80–0.87) in solid tumors (Zou et al., 2020). Taken together, these findings indicate that CDR1as might become an easily detectable prognostic factor for cancer patients. However, our knowledge on the roles of CDR1as in cancer is still limited and further studies are needed. The available reports mainly focused on the function of CDR1as as a miRNA sponge, but circRNAs can also bind to proteins to exert their function. Hence, the other possible functions of CDR1as in cancer should also be investigated. Importantly, various studies showed that CDR1as exerts a vital role in cancer development, and we hope that therapies targeting CDR1as could be applied in the clinical treatment of cancer patients.

## Author Contributions

RS, WW, and JL collected the related manuscript. CJ and XZ drafted and revised the manuscript. RW designed the review. JT and LL participated in the design of the review and helped to draft and revise the manuscript. All authors read and approved the final manuscript.

## Conflict of Interest

The authors declare that the research was conducted in the absence of any commercial or financial relationships that could be construed as a potential conflict of interest.
